# Advancing chirality analysis through enhanced enantiomer characterization and quantification via fast Fourier transform capacitance voltammetry

**DOI:** 10.1038/s41598-023-43945-7

**Published:** 2023-10-05

**Authors:** Mehrnaz Ebrahimi, Parviz Norouzi, Jahan B. Ghasemi, Ali Akbar Moosavi-Movahedi, Meissam Noroozifar, Razieh Salahandish

**Affiliations:** 1 Chemistry Faculty, School of Sciences, University of Tehran, POB 14155-6455, Tehran, Iran; 2 Laboratory of Advanced Biotechnologies for Health Assessments (Lab-HA), Lassonde School of Engineering, York University, Toronto, M3J 1P3 Canada; 3 Department of Electrical Engineering and Computer Science, York University, 4700 Keele Street, Toronto, ON M3J 1P3 Canada; 4 Institute of Biochemistry and Biophysics, University of Tehran, Tehran, Iran; 5 Department of Physical and Environmental Sciences, University of Toronto Scarborough, 1265 Military Trail, Toronto, ON M1C 1A4 Canada

**Keywords:** Analytical chemistry, Electrochemistry, Biomedical engineering, Machine learning

## Abstract

The exploration of the chiral configurations of enantiomers represents a highly intriguing realm of scientific inquiry due to the distinct roles played by each enantiomer (D and L) in chemical reactions and their practical utilities. This study introduces a pioneering analytical methodology, termed fast Fourier transform capacitance voltammetry (FFT-CPV), in conjunction with principal component analysis (PCA), for the identification and quantification of the chiral forms of tartaric acid (TA), serving as a representative model system for materials exhibiting pronounced chiral characteristics. The proposed methodology relies on the principle of chirality, wherein the capacitance signal generated by the adsorption of D-TA and L-TA onto the surface of a platinum electrode (Pt-electrode) in an acidic solution is harnessed. The capacitance voltammograms were meticulously recorded under optimized experimental conditions. To compile the final dataset for the analyte, the average of the FFT capacitance voltammograms of the acidic solution (without the presence of the analyte) was subtracted from those containing the analyte. A distinct arrangement was obtained by employing PCA as a linear data transformation method, representing D-TA and L-TA in a two/three-dimensional space. The outcomes of the study reveal the successful detection of the two chiral forms of TA with a considerable degree of precision and reproducibility. Moreover, the proposed method facilitated the establishment of two linear response ranges for the concentration values of each enantiomer, spanning from 1 to 20 µM, and 50 to 500 µM. The respective detection limits were also determined to be 0.4 µM for L-TA and 1.3 µM for D-TA. These findings underscore the satisfactory sensitivity and efficiency of the proposed method in both qualitative and quantitative assessments of the chiral forms of TA.

## Introduction

The synthesis, separation, and detection of chiral compounds pose significant challenges and are widely recognized as crucial endeavors in diverse scientific fields including drug design, pharmaceutical chemistry^1,2^, material science^3,4^, food technology^5,6^, diagnostics^7,8^, and biomedicine^9,10^. Tartaric acid (TA) serves as an exemplary organic acid found in fruits, tamarinds, and citrus, and finds broad applications as an acidification agent and antioxidant in the pharmaceutical and food industries^11–14^. With its chirality characteristics, TA exists in two enantiomeric forms, namely D-TA and L-TA, which possess distinct functions such as acting as a resolving agent in organic synthesis and inducing carbon dioxide release through interaction with sodium bicarbonate via oral administration, respectively^15,16^.

Various methods have been reported for the recognition of chiral compounds, including chromatographic and spectral-based approaches. In the case of TA, mass spectrometry coupled with vibrational spectroscopy^1^, gas chromatography^17^, liquid chromatography-mass spectrometry^18^, nano-liquid chromatography^19^, and liquid chromatography^20^ have been reported as techniques for its analysis. However, these methods are often considered complex, multi-step, expensive, and time-consuming. Consequently, there is a pressing need to develop simpler, cost-effective, rapid, precise, and highly selective strategies for chiral compound detection. Electrochemical sensors, particularly the modern classified ones have sparked intense interest because they not only enable the detection and quantification of non-electroactive species (such as TA), but also exhibit lower detection limits and broader linear response ranges due to the implementation of specialized signal processing techniques that enhance their sensitivity^21–30^. Nonetheless, it is crucial to address challenges encountered by these contemporary electrochemical techniques, such as random systematic noise, which can considerably impact signal reliability and reproducibility. In this context, signal processing strategies assume importance as they offer notable advantages, including noise reduction and modification of the obtained data for improved validation. Among the potential choices, the Fast Fourier transform (FFT) electrochemical methods stand out as the most suitable candidate.

FFT data processing has been recognized for its capability to effectively eliminate incidental noise in measurements by enabling the transformation of signals between the time domain and frequency domain^31,32^. Through Fourier transformation, the signal-to-noise ratio can be significantly improved, and in certain cases, the faradaic and non-faradaic components of the signal can be extracted from the electrode response. Consequently, the application of electrochemical analysis is not limited solely to electroactive compounds but can also encompass non-electroactive species such as TA, which represents a remarkable and distinctive chirality feature. The complexity of the data obtained from FFT voltammetry necessitates the utilization of machine learning (ML) techniques to establish a comprehensive analysis and interpretation of the acquired qualities.

ML, a prominent branch of artificial intelligence (AI), is widely recognized as a valuable tool for data processing and can be effectively applied in this context^33–35^. ML techniques, including principal component analysis (PCA), principal least squares (PLS), and others, offer a range of statistical approaches to construct computer models capable of extracting information from datasets without the need for explicit programming^36,37^. In essence, ML enables the optimization of experimental conditions and the development of methods for categorizing and distinguishing analytes based on their physical, chemical, or biochemical properties. The field of materials and chemistry has witnessed an increasing interest in the application of ML in recent years^38,39^.

**Figure 1:**
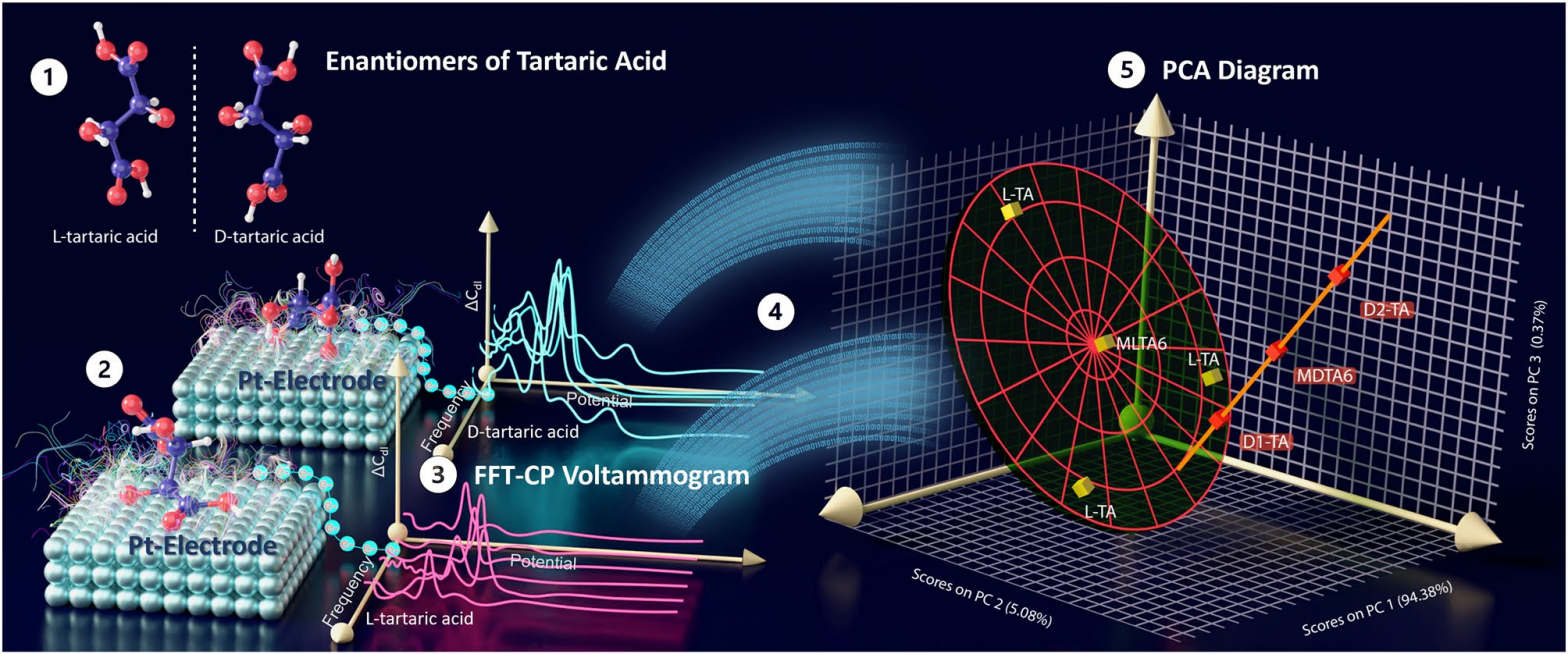
Schematic illustration depicting the key procedural steps of a novel electrochemical approach, namely fast Fourier transform capacitance voltammetry (FFT-CPV), in conjunction with principal component analysis (PCA), for the discernment of chiral enantiomers. The workflow encompasses the following stages: (1) Identification and quantitative determination of the chiral forms, of tartaric acid (TA), D-TA and L-TA, utilizing (2) a platinum electrode (Pt-electrode) immersed in an acidic solution to (3) elicit a capacitance signal generated by the adsorption of D-TA and L-TA onto the electrode's surface. (4) By applying PCA as a linear data transformation technique, a distinctive spatial arrangement is achieved, visually representing D-TA and L-TA in a three-dimensional coordinate system.

In this proof-of-concept study, a cutting-edge analytical technique known as FFT capacitance voltammetry (FFT-CPV), was employed as an effective approach to characterize the chiral forms of TA both qualitatively and quantitatively by utilizing the electrode capacitance at different potentials. FFT-CP voltammograms of D-TA and L-TA were acquired in a sulfuric acid solution using a platinum electrode (Pt-electrode). The capacitance voltammograms of the sulfuric acid were subtracted from those of D-TA and L-TA, resulting in specific voltammetry profiles for each chiral form of TA. The comprehensive electrochemical dataset obtained was subjected to PCA. Notably, the results demonstrated a reasonable level of precision and reproducibility, leading to the identification of distinct regions corresponding to the chiral forms of TA. The proposed method exhibited the ability to detect and separate the two chiral forms of TA, and under optimized experimental conditions, enabled the determination of both forms of TA with relative limits of detection (LOD) values (Fig. 1).

## Experimental

### Fabrication of ME and sample preparation

A Pt wire (identified diameter (i.d) of. 2 mm) and a Pt-electrode (100 µm in radius, Good Fellow Metals Ltd., UK) were employed as the counter electrode (CE), the working electrode (WE) and an Ag/AgCl as the reference electrode, respectively. The Pt wire was securely sealed within a soft glass capillary through the application of heat, after which it was cut to obtain a disk-shaped surface for the working electrode. Silver epoxy resin was used to establish the electrical connection between the Pt wire enclosed in the glass and another wire, possibly made of copper or another metal, for the external connection (Johnson Matthey Ltd., UK).

The utilization of the electrode was based on the observation that it could effectively reduce the high charging current typically associated with high-speed excitation potentials. Additionally, its small surface area facilitated easier cleaning, ensuring a reproducible response. However, it is crucial to maintain reproducibility in this type of measurement. To achieve this, two potential steps were applied at the beginning of the square pulse (SP) ramp on the electrode surface in this experimental approach. The electrochemical surface was cleaned before and after each run using the following steps:

- The Pt-electrode was immersed in Piranha solution (a mixture of H_2_SO_4_ (#7664-93-9, Sigma-Aldrich, USA) and H_2_O_2_ (#7722-84-1, Sigma-Aldrich, USA), in a 3:1 ratio, for 3 min.
- Subsequently, the electrode was polished with alumina powder (#GF29729650, Sigma-Aldrich, USA), for 5 min, followed by ultra-sonication in deionized water for 5 min.
- Finally, the Pt-electrode was immersed and subjected to cyclic voltammetry in an H_2_SO_4_ 0.5 M solution, with a potential range of − 300 to 1300 mV and a sweep rate of 20 mV/s for almost 9 min. A two-step potential was applied at the beginning of the square pulse ramp. The FFTCP voltammograms of the electrode were then recorded in an acidic solution (H_2_SO_4_ 0.05 M) under the following experimental conditions: potential range of − 300 to 1300 mV, frequency of 400 Hz, cycle of 8, and amplitude of 15 mV. This process was repeated until completely repeatable voltammograms were obtained.

The cleaning process lasted less than 20 min and finally, FFT-CP voltammograms were recorded for individual solutions of the analyte D-TA and L-TA (each at a concentration of 10 µM) under the same experimental conditions. Each run was completed in less than a minute. The aforementioned steps were also repeated for subsequent runs.

### The electrochemical setup and methodology

The electrochemical measurements were conducted using a custom-designed potentiostat, as illustrated in Fig. S1. The software was developed using Delphi 6.0 to facilitate data collection, processing, potentiostat control, and potential waveform generation. In essence, the electrochemical setup comprised a computer equipped with a data acquisition board (PCL-818PG, PC-Lab Card Co.) and a potentiostat specifically designed for this purpose (Fig. S1). The circuit consists of two sections: Section (a) encompasses the components connected to the working, counter, and reference electrodes, along with the gain circuit responsible for amplifying the working current output through DG508. The gain circuit offers four amplification levels, with an order of magnitude of 10.

In section (b), a series of operational amplifier (Op-Amp) circuits are present, providing eight-level analog filtration for the signal output. However, it should be noted that these circuits are primarily suitable for low potential scan rates. At high potential frequencies or potential scan rates, their effectiveness may be limited.

The excitation potential waveform and current sampling method employed in the FFT-CPV technique share similarities with the classical method. Figure S2 illustrates the waveform of the square wave (SW), which consists of multiple SW pulses (referred to as polarization cycles, *N*_*c*_) ranging from 2 to 32. Each SW pulse has an amplitude (E_sw_) ranging from 5 to 30 mV and is superimposed on a staircase potential function with a small potential step (ΔE) of 10 mV. However, in FFT-CPV, there is a notable difference. The currents are sampled eight times per cycle of SW polarization, resulting in an increased number of data points available for averaging and capturing the contributions of non-faradaic currents. This augmented data collection enhances measurement precision and supports the subsequent FFT calculation, which involves the computation of the electrode capacitance value.

In terms of the electrochemical setup, it includes an Ag/AgCl reference electrode (RE), a platinum wire with a 2 mm inner diameter serving as the CE, and a Pt-electrode acting as the WE.

A succinct overview of the fundamental principles underlying the data treatments employed in FFT-CPV is provided in the Supplementary Information, SI-1, while a comprehensive discourse on these principles can be found in the report by Sluyters-Rehbach and Sluyters^40^.

### PCA model

The machine learning model of PCA serves as an established technique for exploring the structure of a dataset, identifying patterns, discerning sample distribution in multivariate space, and detecting relevant parameters within that space. PCA operates on the fundamental principle of transforming a set of correlated variables into a new set of uncorrelated variables known as principal components (PCs). This process involves decomposing the data matrix, denoted as X, into distinct components representing the essential information and noise fragments, as represented by Eq. S7.

Preprocessing procedures were applied to the raw electrochemical dataset prior to subjecting it to machine learning algorithms. The dataset was transformed into a data matrix, denoted as X_n×m_, where n represents the number of signal traces and m represents the number of potential steps. The preprocessing operations consisted of two steps: the application of Standard Normal Variate (SNV) transformation and the utilization of a second derivative function of Savitzky-Golay with a window size of 13 points and a degree 3 polynomial for smoothing^41^.

For quantification purposes, the data matrix X was imported into MATLAB. Noisy and flat regions were visually identified and excluded from further analysis as they lacked informative content. The data matrix X was then mean-centered and autoscaled. Mean-centering removed any background signal that was not dependent on the concentration of the analytes, while autoscaling eliminated variables with high standard deviations that could potentially hinder variables with low variation.

This normalization procedure aimed to ensure a balanced representation of variables and mitigate the influence of outliers, thereby enhancing the reliability of subsequent analyses. The selection of the most efficient and optimal model relied on evaluating the root-mean-standard error for calibration (RMSECV). Models were assessed based on their ability to minimize RMSECV, with lower values indicating superior performance and a higher degree of accuracy in calibration.

## Results and discussion

### FFT-CPV parameters optimization

**Figure 2:**
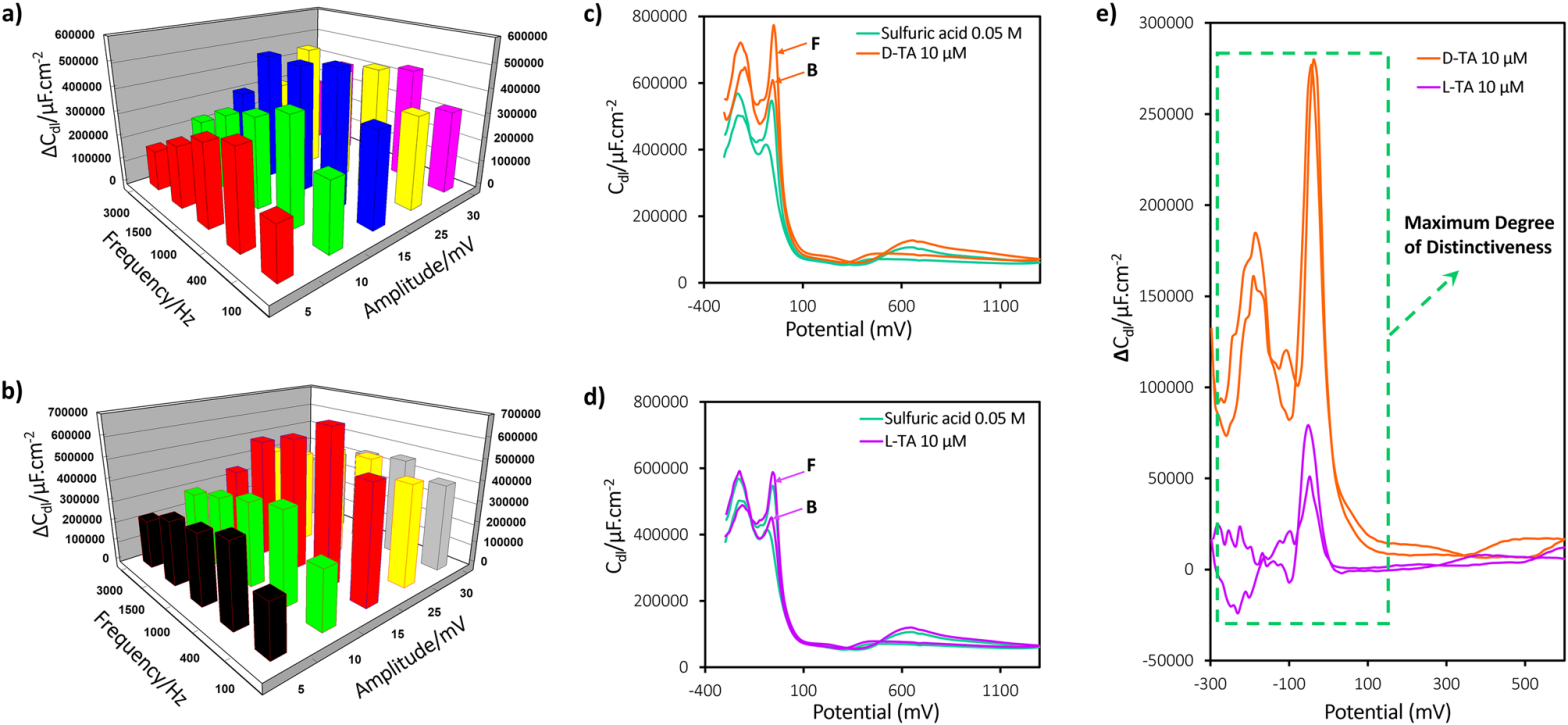
Optimization of FFT-CPV parameters and FFT-CPV patterns serve as distinctive signatures for the characterization of two enantiomers of TA with different chiral forms (D and L). The optimization of frequency and amplitude for FFT-CPV analysis in an acidic solution (H_2_SO_4_ 0.05 M) with a Pt-electrode vs. Ag/AgCl reference electrode over the specified ranges: Frequency of 100 to 3000 Hz, amplitude of 5 to 30 mV, at the number of cycles of pulses of 8, and potential range of − 300 to 1300 mV for (**a**) D-TA, and (**b**) L-TA. The FFT capacitance signal of (**c**) D-TA 10 µM, (**d**) L-TA 10 µM obtained in the H_2_SO_4_ 0.05 M, and (**e**) the differential capacitance (∆C_dl_) voltammograms of each enantiomer of TA derived from H_2_SO_4_ 0.05 M, conducted within the optimum frequency of 400 Hz, 8 cycles, and amplitude of 15 mV at the surface of Pt-electrode. To ensure accuracy and reliability, all experiments were repeated three times (n = 3), and the presented plots represent the average of the results. The relative standard deviation (RSD) for the obtained data was found to be < 3%. F and B indicate forward and backward scans of potential, respectively.

To ensure the highest performance efficiency of the method for both qualitative and quantitative measurements, it is essential to record FFT-CPV voltammograms under optimal conditions. Within the methodology section, several parameters are involved in FFT-CPV, including the frequency (*f*_*0*_), the number of cycles of pulses (*N*_*c*_) in each potential step (ΔE) of the ramp, and the amplitude (*E*_*sw*_). To optimize the *f*_*0*_ parameter, the FFT-CP signal of the electrode was recorded in an acidic solution (H_2_SO_4_ 0.05 M) within a potential range of − 300 to 1300 mV, while varying frequencies from 100 to 3000 Hz. Additionally, *E*_*sw*_ was investigated in the range of 5 to 30 mV. The FFT-CPV signal for each enantiomer of TA, after baseline correction, was then plotted against the frequency and amplitude values, as illustrated in Fig. 2a and b. Upon analyzing the plot for both analytes, the optimized values for frequency and amplitude were determined as 400 Hz and 15 mV, respectively. When *N*_*c*_ was held constant at 8, the potential rate increased with frequency. It was observed that the FFT-CPV signal (or current) exhibited enhancement up to 800 Hz. Likewise, increasing the SW amplitude (up to 15 mV) led to a faster excitation potential (larger value of dE/dt) and a higher FFT-CPV signal. However, at higher frequencies (or higher SW amplitudes), a decline in the FFT-CPV signal was observed due to diffusion limitations for the analyte towards the electrode surface. For a comprehensive understanding of the differential capacitance voltammograms, including their variations with different amplitudes and frequencies for both D-TA and L-TA, refer to the SI-2 section, Figs. S3 and S4, respectively.

In another aspect of the optimization process, *N*_*c*_ was systematically varied from 2 to 32 pulses, which proved to be a crucial factor affecting the potential scan rate. While maintaining a constant *f*_*0*_ of 400 Hz and *E*_*sw*_ of 15 mV, it was observed that the potential scan rate decreased as *N*_*c*_ increased. Specifically, when *N*_*c*_ was increased up to 8, a noticeable enhancement in the FFT-CP signal was observed for both cases, as illustrated in Figs. S5 and S6. Notably, for *N*_*c*_ values lower than 8, the potential excitations rate exceeded the limitations imposed by the electrode processes, resulting in suboptimal performance. However, beyond this value, the overall scan rate started to decline, subsequently leading to a decrease in the magnitude of the signal.

### Electrochemical dataset generation

Initially, FFT-CP voltammograms were recorded using a Pt-electrode in the acidic solution, with a frequency of 400 Hz, an amplitude of 15 mV, and *N*_*c*_ set at 8. Subsequently, under the same experimental conditions, the electrode response in the presence of the analyte solution (D-TA and L-TA, each at a concentration of 10 µM) was recorded, separately. Figure 2c and d illustrate the FFT-CP voltammograms of the D and L enantiomers of TA, as well as the FFT-CP voltammogram of the pure acidic solution. A slight difference can be observed between the voltammograms in each graph.

To obtain the differentiated voltammograms (∆C_dl_), the FFT-CP voltammograms of the two analytes (D-TA and L-TA) were subtracted from the FFT-CP voltammogram of the acidic solution following the equation:

1 $$\Delta {\text{C}}_{{{\text{dl}}}} = {\text{ C}}_{{{\text{dl}}}} \left( {{\text{analyte}}} \right) \, - {\text{ C}}_{{{\text{dl}}}} \left( {{\text{acid}}} \right)$$

The differences in ∆C_dl_ values observed in the FFT-CPVs of sulfuric acid solutions containing D-TA or L-TA stem from the enantiomeric structures of the two analytes. To examine the physical phenomenon underlying these differences, it is important to consider the interaction of the enantiomers with the electrode surface. Enantiomers are mirror images of each other, and their distinct three-dimensional structures can lead to different interaction patterns and surface coverage at the electrode interface. This includes variations in how they adsorb, orient, and interact with solvent molecules and the electrode surface itself. In fact, when an enantiomer adsorbs onto the electrode surface, it creates a unique arrangement that influences the double-layer charge distribution or ∆C_dl_^42–44^. This, in turn, leads to changes in the double-layer capacitance. The resulting diagrams are presented in Fig. 2e. As observed, there exists a discernible distinction between the response regions of D-TA and L-TA, with the red rectangle demarcating the maximum level of differentiation. This finding indicates that these specific regions hold the potential for selective measurements of D-TA and L-TA. However, it is important to note that the signals still exhibit the possibility of further differentiation between D-TA and L-TA. Consequently, the data underwent further analysis using PCA to explore additional discriminatory information. To uphold the scientific rigor and validity of the findings, the experimental procedures were meticulously replicated in triplicate. The relative standard deviation (RSD) of the acquired data was computed and found to be below 3%, underscoring the consistency and reliability of the measurements.

### Chemometrics analysis of the electrochemical dataset

The PCA method was employed to examine the distribution pattern of TA chiral forms and quantify them using the FFT-CPV outcomes. The overall workflow and related steps are summarized and presented in section SI-3, Fig. S7. In the preliminary PCA, it was observed that some variables (potentials) with absolute loadings smaller than 0.2 contributed to the deterioration of the Euclidean distance between the two groups or clusters corresponding to the expected analytes (enantiomers) in the PC space. Consequently, those variables were excluded from further analysis.

The potential range selected by the model encompassed a vast majority of electrochemical profiles, with a notable concentration of viewers falling within the range of − 300 to 1300 mV. By eliminating a specific set of values from a pool of 17 × 333, data reduction was achieved without compromising the model's integrity, performance, and ability to differentiate the data effectively.

**Figure 3:**
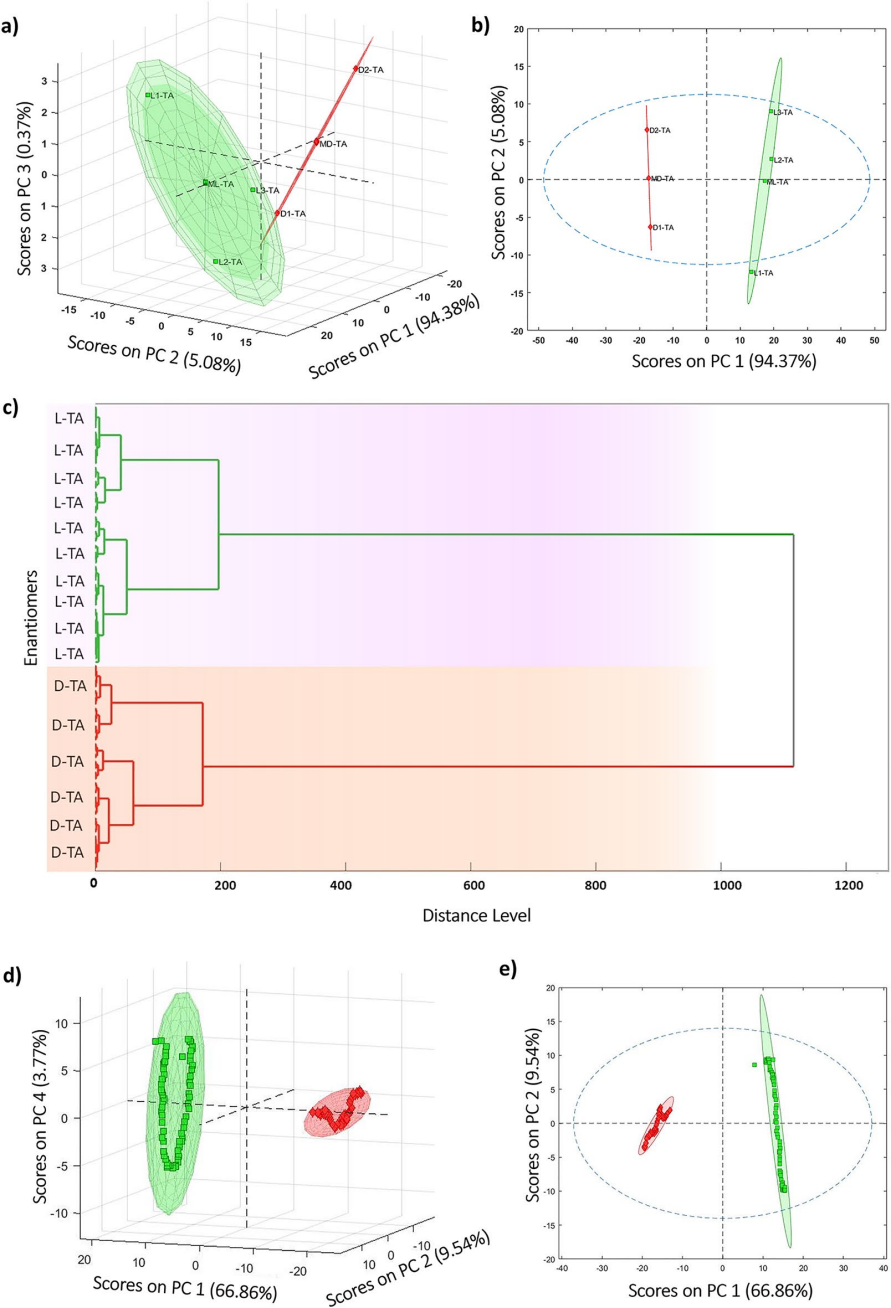
TA enantiomers differentiation based on their FFT capacitance signal. (**a**) 3D representation, demonstrating the application of PCA to the electrochemical dataset enabling accurate pattern recognition of D-TA and L-TA, and (**b**) a 2D format, providing a clear visualization of the distinctive patterns exhibited by the enantiomers. ML-TA and MD-TA stand for the mean of L-TA and D-TA samples respectively with 6 replications. (**c**) The outcome of the clustering analysis conducted on the PCA-analyzed dataset. (**d**) The 3D and (**e**) 2D representations of PCA diagram after introducing noise ranging from 0 to 0.2% to the electrochemically created dataset using the Monte Carlo method, providing insights into the robustness of the analysis even in the presence of noise.

The score-score plots of the first three and two principal components are shown in Fig. 3a and b, respectively. These plots reveal two distinct clusters, indicating the separation of the two enantiomers along the PC1 direction. The PC2 direction accounts for within-cluster variations and explains approximately 5% of the total variance.

To assess the number of different groups in the electrochemical traces, a dendrogram was constructed using hierarchical cluster analysis (HCA), as shown in Fig. 3c. The dendrogram highlights the presence of two groups with a significant distance between them.

To demonstrate the robustness of the technique in detecting and identifying enantiomers from both the original and noise-added traces, a PCA model was built and repeated multiple times using resampled profiles generated by the Monte Carlo method. This method introduced noise ranging from 0 to 0.2% to the electrochemically created dataset. The results of this analysis, presented in both 3D and 2D formats in Fig. 3d and e, reaffirm the separation of the two clusters along the PC1 direction, thus confirming the method's efficacy in efficiently identifying the two enantiomers.

To demonstrate the versatility of the proposed method for the mixture solutions of the L-TA and D-TA, simulations involving typical mixtures created by linearly combining the pure profiles of the two enantiomers were conducted. In this approach, an initial solution containing the pure D-TA enantiomer was utilized, followed by intermediate solutions representing linear mixtures of both enantiomers. Finally, the pure form of the L-TA enantiomer was included in the last solution. Following this rationale, the mixture traces were prepared without any data manipulation, and the resulting data matrix was subjected to standard PCA. The transformation of objects/solutions into the new space is illustrated in Fig. S8. As shown in the figure, adhering to the principles of linear combinations, it becomes apparent that all samples are aligned along a single line, resulting in the detection of only one PC. This is attributed to the fact that when the sum of X1 and X2 equals 1, the degree of freedom is reduced to one, leading to a matrix rank of 1. Consequently, PC1 alone can adequately account for all variations in the data matrix.

Overall, the combination of PCA, score-score plots, dendrogram analysis, and repeated model building using noise-added traces demonstrate the capability of the technique to successfully detect and identify the enantiomers from the original and noise-introduced electrochemical data.

### Quantification analysis

**Figure 4:**
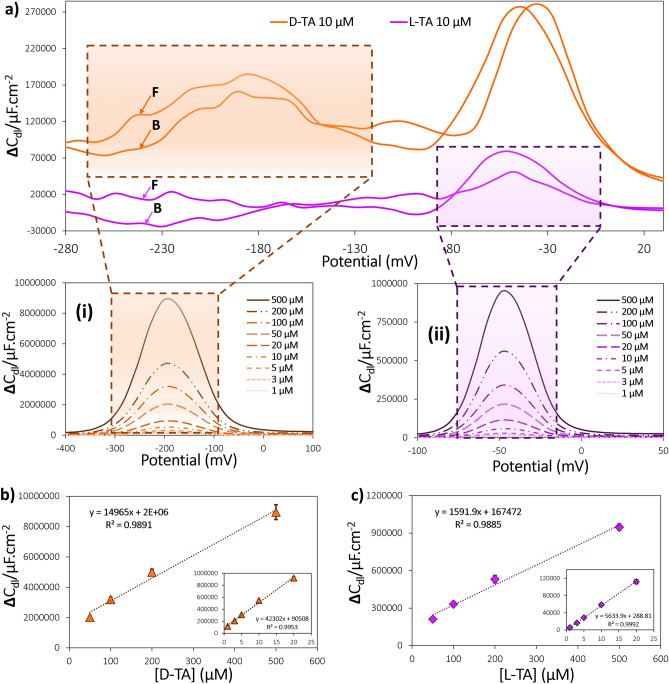
The differentiated and baseline-corrected FFT-CPV accompanied by the obtained analytical figures of merit. The voltammogram of (**a-i**) D-TA and (**a-ii**) L-TA at different concentrations and (**b**) and (**c**) the corresponding calibration curves for D-TA and L-TA, respectively, demonstrating the dynamic range of 1 to 500 µM under the optimum experimental conditions, including a potential range of − 300 to 1300 mV, a frequency of 400 Hz, 8 cycles, and an amplitude of 15 mV at the Pt-electrode surface vs. Ag/AgCl reference electrode. F and B indicate forward and backward scans of potential, respectively.

In the subsequent phase, the performance of the Pt-electrode was assessed for the quantification of D- and L-TAs. The selection of the peak and appropriate signal processing techniques were crucial for obtaining accurate calibration data. Initially, all peaks in the FFT-CPV voltammograms were examined to identify the peak with the best sensitivity or signal-to-noise ratio. The selected peak for each analyte was then extracted from their respective voltammograms. The applied signal processing techniques included digital filtration using the FFT method, background subtraction, and correction for drift removal. The resulting processed signal is shown in Fig. 4a. In this figure, the selected peaks for the determination of D-TA and L-TA are located at − 200 mV and − 50 mV, respectively.

To evaluate the sensor's performance, solutions of D-TA and L-TA with concentrations ranging from 1 µM to 1 mM were carefully evaluated. The capacitance signal of each solution was recorded under controlled parameters, including a frequency of 400 Hz, 8 cycles, and an amplitude of 15 mV, within a potential range of − 300 to 1300 mV (Fig. 4a-i and -ii). The obtained FFT capacitance voltammograms were then subjected to baseline correction and plotted against their corresponding concentration values.

The resulting calibration curves, shown in Fig. 4b and c, highlight the sensor's performance with respect to dynamic range and sensitivity. It is important to note that the calibration curve is not linear over a wide range of concentrations due to the nature of the detection processes, which rely on the electrochemical adsorption of the analyte. At low concentrations, the adsorption process follows the Langmuir adsorption isotherm model, while at higher concentrations, other models such as the Freundlich adsorption isotherm model come into play. Therefore, there are two linear concentration ranges for the calibration curve. For D-TA, the sensor exhibited a sensitivity of 42,302 µF L cm^−2^ µmol^−1^ within the dynamic range of 1 to 20 µM, and a sensitivity of 14,965 µF L cm^−2^ µmol^−1^ within the dynamic range of 50 to 500 µM. Similarly for L-TA, the sensor showed a sensitivity of 5634 µF L cm^−2^ µmol^−1^ within the dynamic range of 1 to 20 µM, and a sensitivity of 1592 µF L cm^−2^ µmol^−1^ within the dynamic range of 50 to 500 µM. The LoD was determined to be 0.4 µM for D-TA and 1.3 µM for L-TA.

Furthermore, the limit of quantification (LOQ) for these enantiomers was also found to be 1.4 µM and 4.4 µM for D-TA and L-TA, respectively. The reliability and accuracy of the presented sensor were confirmed by the absence or minimal overlap observed between the error bars associated with different measured concentrations of D-TA and L-TA within the calibration curve. Table S1 provides a comparative analysis of the performance metrics in our study in relation to recent investigations aimed at the quantification of TA.

## Conclusions

The investigation of chiral enantiomers bears significant importance due to their distinct roles in chemical reactions and practical applications. This study demonstrated the efficacy of FFT-CPV coupled with PCA as a powerful electroanalytical technique for the precise identification and quantification of tartaric acid's enantiomeric forms. This new method emerged as a promising EC detector for the direct measurement of a large number of non-electroactive compounds and analternative to the time-intensive EIS measurements commonly used for sensor characterization. The proposed method effectively generated unique fingerprints for each enantiomer by employing a non-faradic electrochemical approach tailored for the non-electroactive nature of TA. FFT-CPV in conjunction with PCA enabled accurate identification and differentiation of chiral isomers, even in cases of high structural similarity. The chiral adsorption process on a Pt-electrode surface in an acidic solution served as a basis for meticulously recording capacitance voltammograms under optimized experimental conditions. Moreover, the proposed method facilitated the establishment of two linear response ranges spanning from 1 to 20 µM and 50 to 500 µM for both enantiomers. The detection limits for D-TA and L-TA were determined to be 0.4 µM and 1.3 µM, respectively, emphasizing the method's satisfactory sensitivity and efficiency for qualitative and quantitative assessments of TA's chiral forms. In summary, the combination of FFT-CPV, PCA, and the property of chiral isomers has introduced novel avenues for the separation and analysis of structurally similar enantiomers. The proposed method offers a precise, rapid, specific, and user-friendly approach for determining chiral isomers, exhibiting considerable potential in various applications within the realms of chiral analysis and the pharmaceutical industry especially when one form of the synthesized ingredient is toxic.

### Supplementary Information

Supplementary Information.

## Data Availability

The datasets analyzed during the current study are available from the corresponding authors upon reasonable request.
