# Supplementary material for: Advancing chirality analysis through enhanced enantiomer characterization and quantification via fast Fourier transform capacitance voltammetry
Source: Sci Rep. 2023 Oct 5;13:16739. doi: 10.1038/s41598-023-43945-7 (PMC10556018; doi:10.1038/s41598-023-43945-7)
Supplement: Supplementary file 1 — Supplementary Information. [file 41598_2023_43945_MOESM1_ESM.docx]

**Supplementary Information**

**Advancing Chirality Analysis through Enhanced Enantiomer Characterization and Quantification via Fast Fourier Transform Capacitance Voltammetry**

Mehrnaz Ebrahimi^1^, Parviz Norouzi^1,2,3^*, Jahan B. Ghasemi^1^, Ali Akbar Moosavi-movahedi^4^, Meissam Noroozifard^5^, Razieh Salahandish^2,3^*

*^1^Chemistry Faculty, School of Sciences, University of Tehran, Tehran POB, 14155-6455, Iran*

*^2^ Laboratory of Advanced Biotechnologies for Health Assessments (Lab-HA), Lassonde School of Engineering, York University, Toronto M3J 1P3, Canada*

*^3^ Department of Electrical Engineering and Computer Science, York University, 4700 Keele Street, Toronto, ON, M3J 1P3, Canada*

*^4^ Institute of Biochemistry and Biophysics, University of Tehran, Tehran, Iran*

*^5^ Department of Physical and Environmental Sciences, University of Toronto Scarborough, 1265 Military Trail, Toronto, Ontario, M1C 1A4, Canada*

**SI-1. Principles of fast Fourier transform capacitance voltammetry (FFT-CPV)**


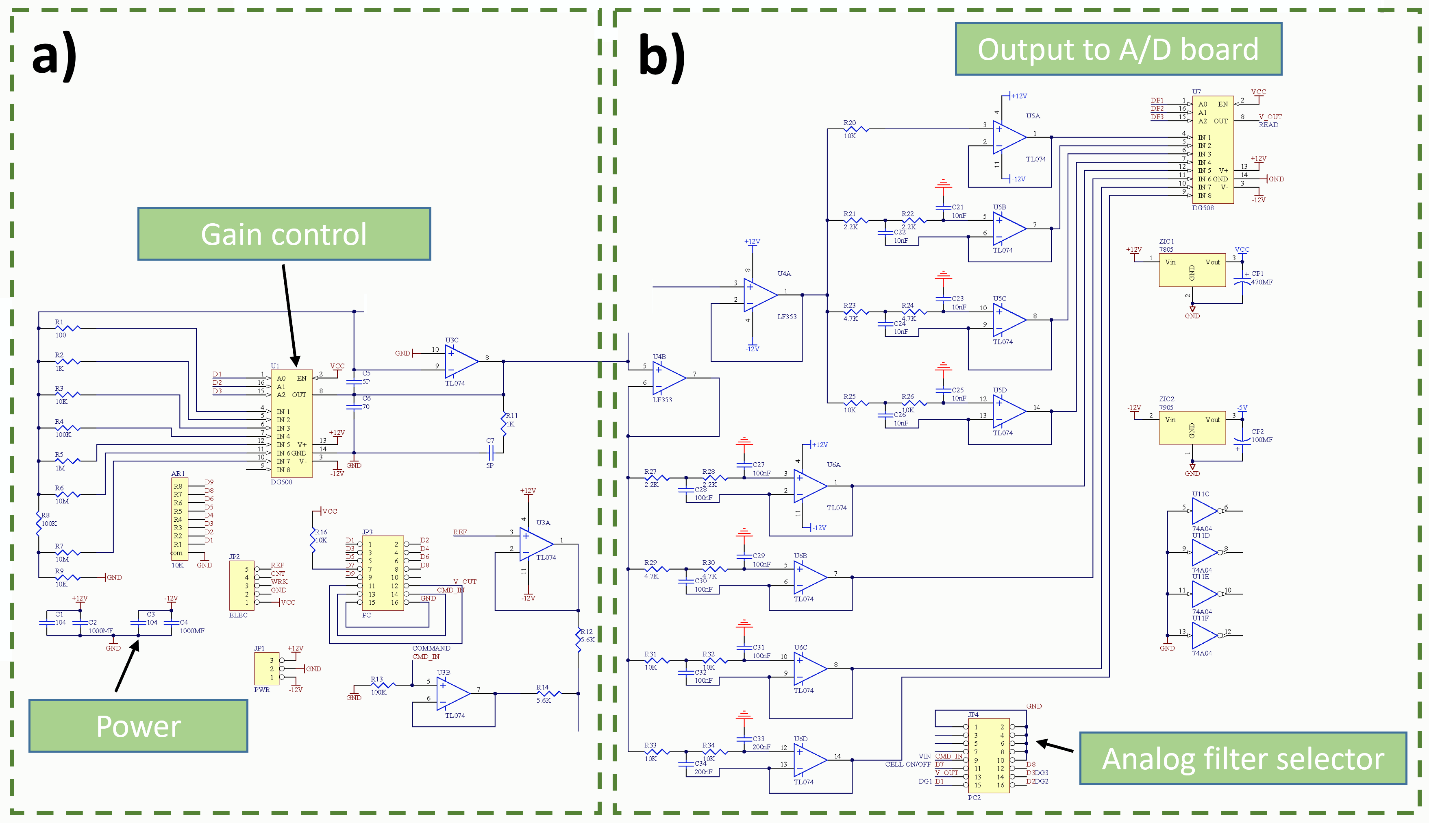


**Figure S1.** Schematic display of the potentiostat circuit utilized for fast Fourier transform capacitance (FFT-CP) measurements, consisting of two distinct parts: (a) and (b). In section (a) various components are interconnected with the working, counter, and reference electrodes, along with the gain circuit responsible for amplifying the working current output through the implementation of (DG508). This section offers seven amplification levels, spanning orders of magnitude around 10. Moving on to section (b), which encompasses analog filtration of the signal output, a series of Op-Amp circuits (eight levels) are available for utilization specifically at low potential scan rates.


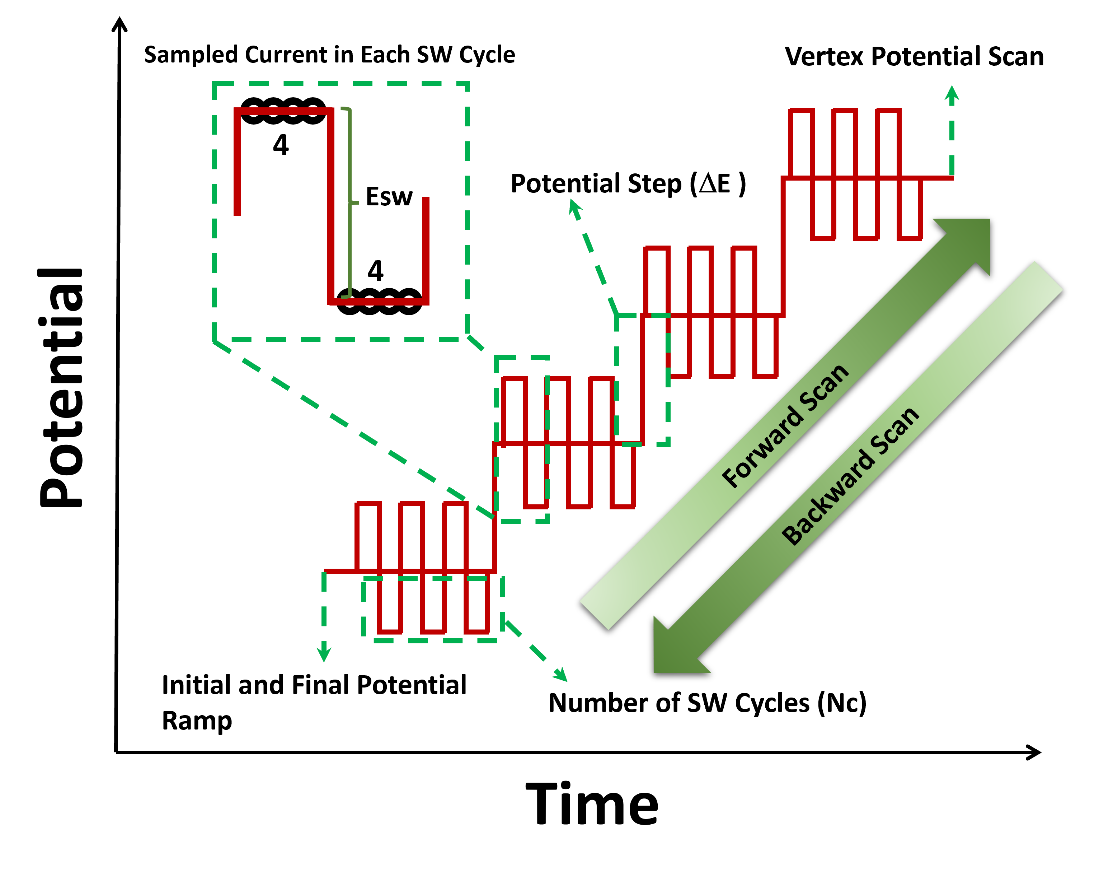


**Figure S2.** Schematic diagram illustrates the applied potential waveform, characterized by the superimposition of multiple square-wave (SW) potentials onto each step of the potential ramp. Additionally, during each SW potential cycle, the currents were sampled eight times. This collection of current data proved crucial in the calculation of both the imaginary and real components of the signal, aiding in the determination of the electrode's capacitance value.

In the FFT-CPV technique, a small square wave (SW) potential, superimposed on a potential ramp, is applied to the system according to the following equation:

$Esq (t)=\Delta E(\omega t)+vt$ Eq. S1

where ΔE is the SW amplitude, ω is the angular frequency of the applied potential waveform (ω = 2πf) and *v* is the potential linear scan rate. The resulting current can be expressed in several harmonics.

$I_{ac}(t)=\Delta I_{1}\cos(\omega t+\phi_{1})+\Delta I_{2}\cos(2\omega t+\phi_{2})+\Delta I_{3}\cos(3\omega t+\phi_{3})+\ldots$ Eq. S2

where ΔI denotes the magnitude of the SW current, φ is its phase shift of the SW current with respect to the applied potential and the subscripts refer to a specific harmonic of the current electrode signal. It should be noted that non-linear systems produce the higher terms in Eq. S2. However, under this experimental condition, the electrode/solution interface could be preserved as a near-liner system in this method. The first term in Eq. 2 is the fundamental signal of the electrode, which defines the signal at the same frequency as the applied potential. The time domain fundamental current can be represented as a sequence of frequency, f_n_ values, resulting in a complex series, according to the discrete Fourier transformation theory.

$I_{o}=\sum_{n=0}^{N-1} F_{n}\cos\left( \frac{2\pi n}{N} \right)+jF_{n}\sin\left( \frac{2\pi n}{N} \right)$ Eq. S3

The currents were sampled at 8 points per cycle, as stated in this method and the discrete Fourier transformation was very simple to apply. The excitation potential and electrode response can then be described as a periodic function and the calculated electrode impedance is:

$Z_{r}(t)=\frac{{8E}_{\omega t}}{I_{0}+I_{1}+I_{2}+I_{3}-I_{4}-I_{5}-I_{6}-I_{7}}$ Eq. S4

and

$Z_{i}(t)=\frac{{8E}_{\omega t}}{{((I}_{0}+I_{1})-{(I}_{2}-I_{3})-({(I}_{4}+I_{5})-{(I}_{6}-I_{7})}$ Eq. S5

where N is the number of data, Z_r_(ωt) and Z_i_(ωt) are the real and imaginary parts of the electrode impedance.

It should be noted that initial changes in electrode capacitance (or the electrode current) occur when the analyte adsorbs to the surface, assuming that the total capacitance is not limited by the solution impedance. Since the proposed technique works on the basis of the species absorbance regardless of their electroactivity, it integrates elements from both SWV and EIS ^1^ and incorporates established mathematical procedures like FFT ^2^ and PCA that such combination has never been used before. However, a direct comparison with commercially available electrochemical systems is still a challenging task due to the current fundamental differences in the method's underlying principles, it could be highlighted that this technique's speed rivals that of SWV, making it practical for various applications. Furthermore, the introduced work offers a fresh perspective and a novel method that has the potential to bridge the gap between traditional electrochemical techniques and advanced chemometric analyses. However, the electrochemical processes may change the value of the obtained current, which may help the specificity of FFT-CPV for each compound. Theoretically, to obtain the highest electrode response the square-wave frequency must be significantly higher than the electrode time constant, where the value of Z_dl_ is then related to capacitance as follows:

$C_{Et}=-\frac{1}{AZi_{\omega t}\omega}$ Eq. S6

where A is the electrode's geometric surface area and C*_Et_* is the electrode's serial capacitance at each potential (or time).

**SI-2. FFT-CPV Parameters Optimization**


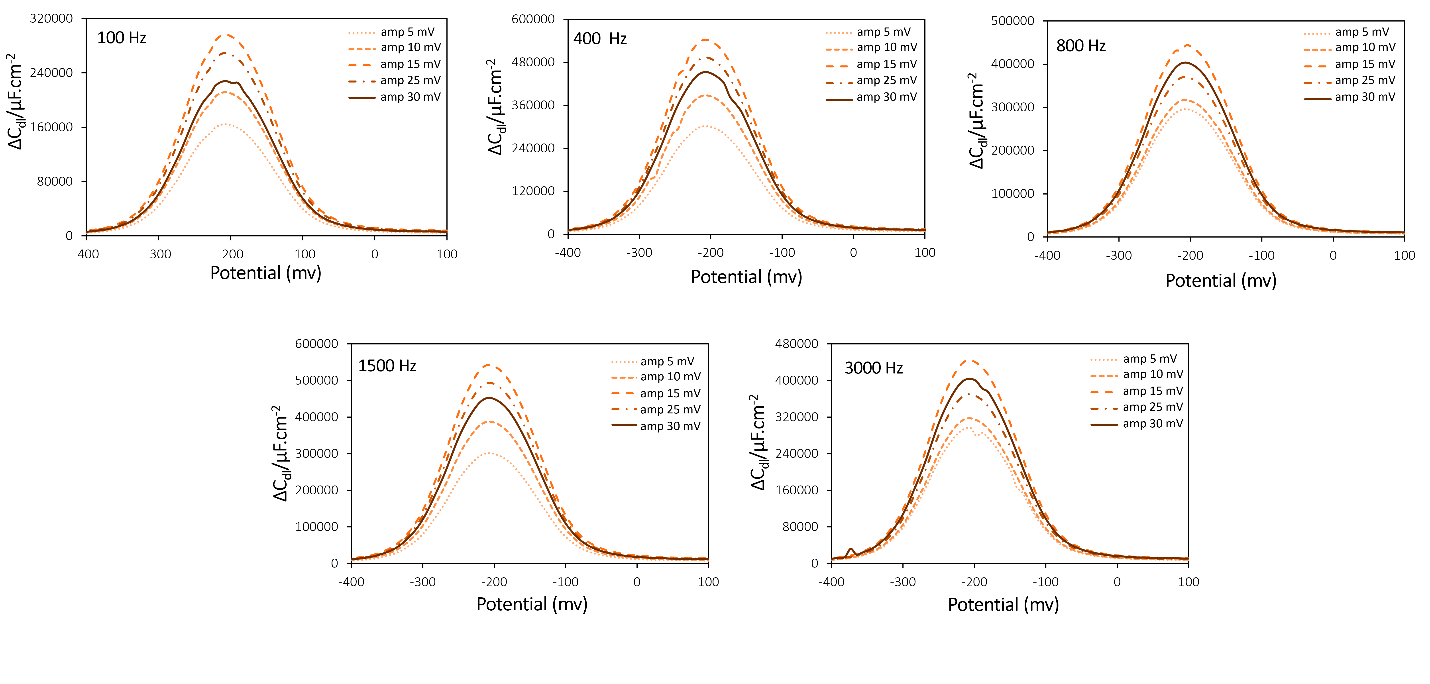


**Figure S3.** The FFT differential capacitance voltammograms obtained during the optimization of frequency and amplitude for FFT-CPV analysis in an acidic solution (H_2_SO_4_ 0.05 M) using a Pt-electrode vs. Ag/AgCl reference electrode, conducted within the following ranges: a frequency range of 100 to 3000 Hz, an amplitude range of 5 to 30 mV, with a fixed number of cycles of pulses set to 8, and a potential range of -300 to 1300 mV for the D-chiral form of tartaric acid (D-TA). The voltammograms were subjected to various data processing steps, including smoothing, background subtraction, and correction to enhance the clarity and accuracy of the obtained data.


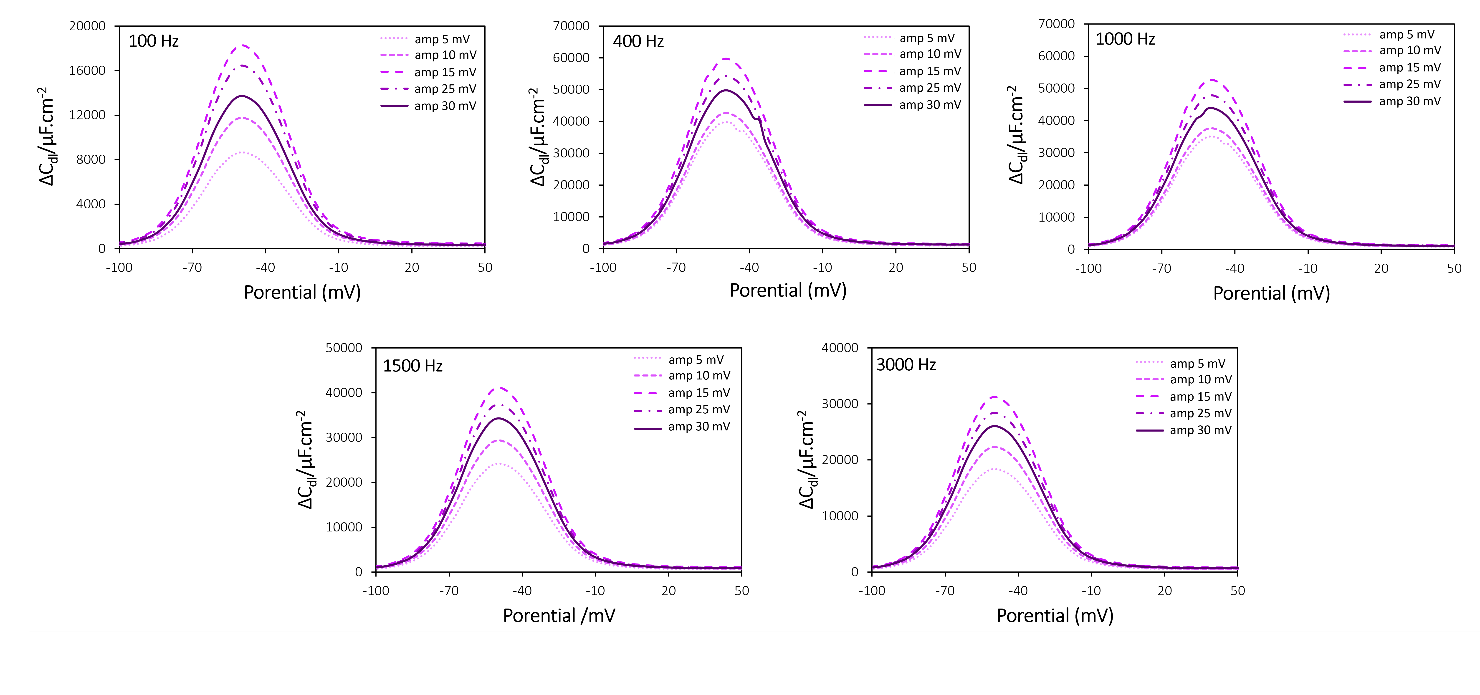
**Figure S4.** The FFT differential capacitance voltammograms obtained during the optimization of frequency and amplitude for FFT-CPV analysis in an acidic solution (H_2_SO_4_ 0.05 M) using a Pt-electrode vs. Ag/AgCl reference electrode, conducted within the following ranges: a frequency range of 100 to 3000 Hz, an amplitude range of 5 to 30 mV, with a fixed number of cycles of pulses set to 8, and a potential range of -300 to 1300 mV for the L-chiral form of tartaric acid (L-TA). The voltammograms were subjected to various data processing steps, including smoothing, background subtraction, and correction to enhance the clarity and accuracy of the obtained data.


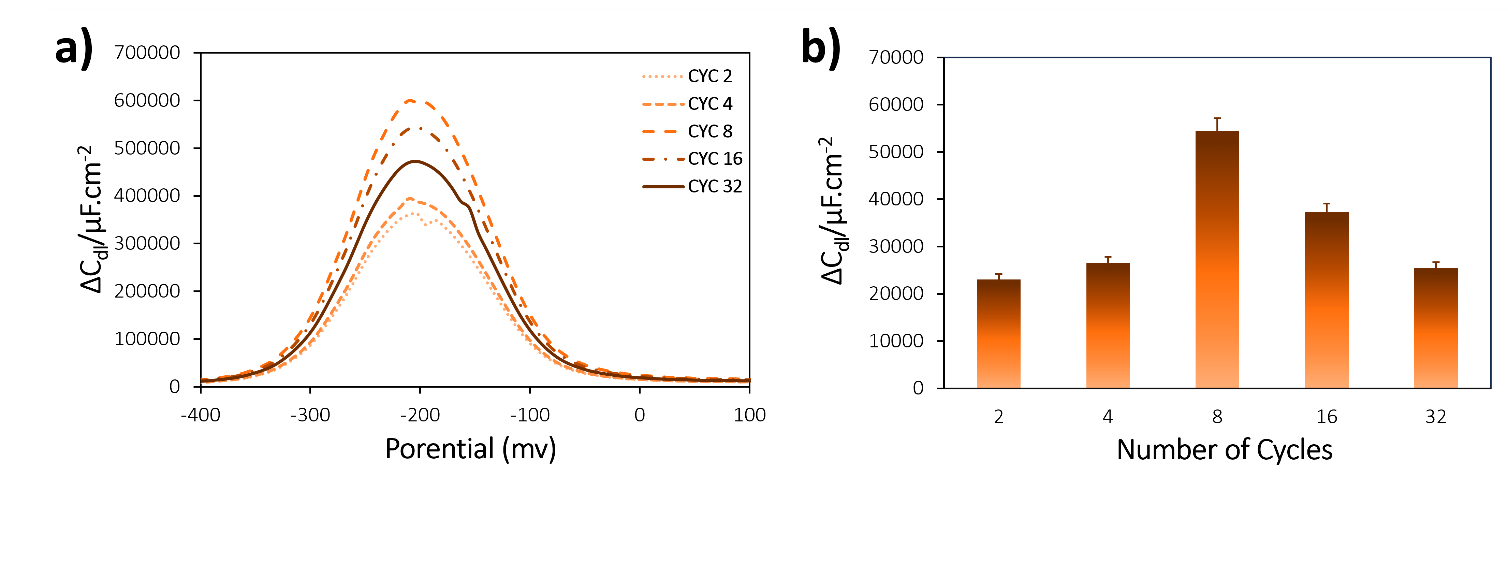


**Figure S5.** (a) The FFT differential capacitance voltammograms obtained for D-TA during the optimization of the number of SW cycles (*N_c_*), providing valuable insights into the capacitance variations observed with different *N_c_* values, and (b) the associated graph, showcasing the optimum *N_c_* in SW frequency of 400 Hz and amplitude of 15 mV.


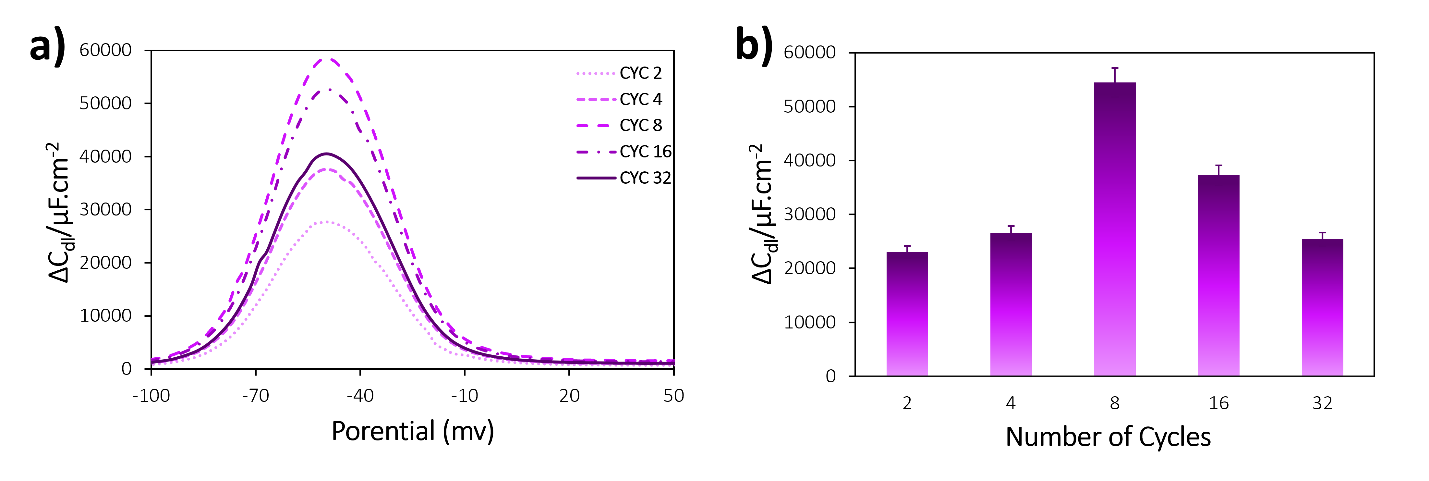
**Figure S6.**  (a) The FFT differential capacitance voltammograms obtained for L-TA during the optimization of the number of SW cycles (*N_c_*), providing valuable insights into the capacitance variations observed with different *N_c_* values, and (b) the associated graph, showcasing the optimum *N_c_* in SW frequency of 400 Hz and amplitude of 15 mV.

**SI-3. Principal Component Analysis (PCA)**

X = TP^T^ + E Eq. S7

Here, X denotes the data matrix comprising measurements or observations, with n rows and m columns representing the number of observations and variables, respectively. The superscript T represents the transpose operation, resulting in a score matrix (n×l) denoted as T, which encompasses the projections of the original data (the X score, component, or factor matrix). Additionally, the orthogonal loading matrix (m×l) is represented by P. Furthermore, the residual matrix, E with I rows and J columns, is assumed to consist of independent and identically distributed random normal variables.


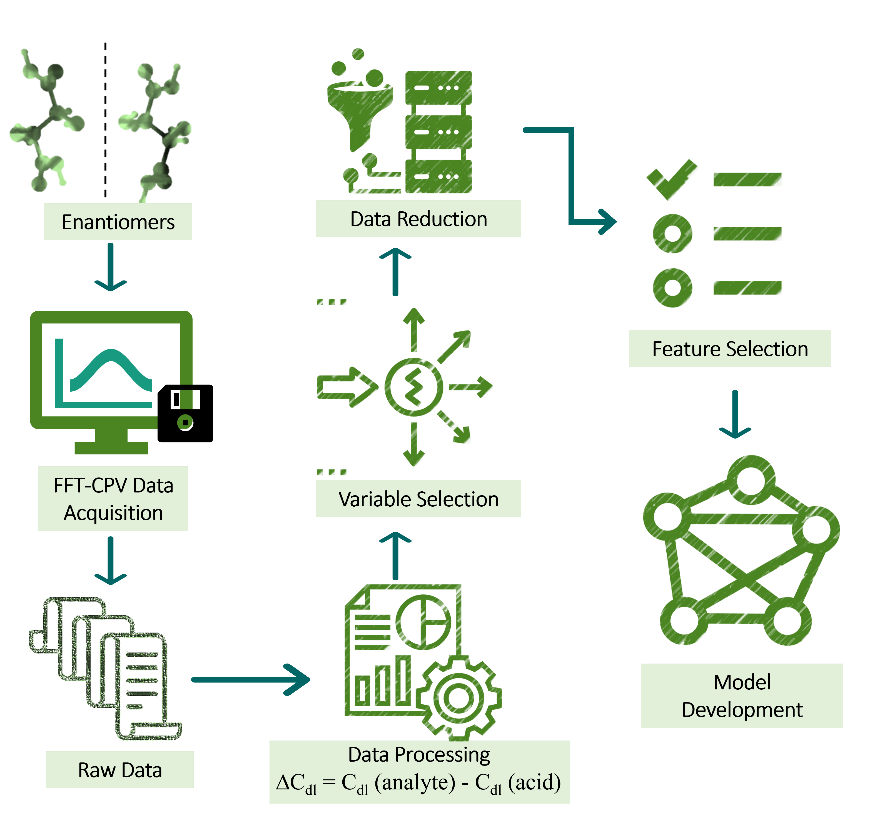


**Figure S7.** Schematic representation outlining the sequential stages involved in principal component analysis (PCA) analysis.


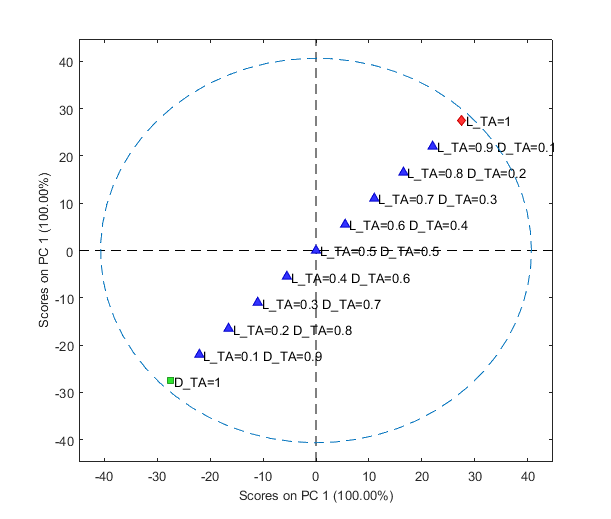


**Figure S8.** The PCA simulations of mixtures of D-TA and L-TA in solution, where created by linearly combining the pure profiles of the two enantiomers.

**Table S1.** Comparison of the proposed method performance and some other recent electrochemical pathways for TA determination.

| No. | Active surface | Method | Linear range | LOD | Ref. |
| --- | --- | --- | --- | --- | --- |
| 1 | Ba bismuthate nanobelts | Cyclic voltammetry | 0.001–2 mM | 0.12 µM | ^3^ |
| 2 | Aluminium bismuthate nanorods modified glassy carbon electrode | Cyclic voltammetry | 0.001–2 mM | 0.64 μM | ^4^ |
| 3 | Polyaniline/bismuth nickelate nanorod composites | Cyclic voltammetry | 0.0005–2 mM | 0.18 μM | ^5^ |
| 4 | molecularly imprinted polymer with gold nanoparticles modified screen-printed electrode | Differential pulse voltammetry | 20.00–227.81 μg/mL | 0.001 μg/mL | ^6^ |
| 5 | Nanosheets of Cu bismuthate | Cyclic voltammetry | 0.008-1 mN | 0.52 μM | ^7^ |
| 6 | CuS-modified glassy carbon electrode | Cyclic voltammetry | 0.005-2 mM | 0.32 μM | ^8^ |
| 7 | Phytalocyanine-Co(II) modified electrode | Differential pulse voltammetry | 10-100 mM | 7.29 μM | ^9^ |
| 8 | **Pt-electrode** | **FFT-capacitance voltammetry** | **1 to 20 µM** | **0.4 µM (D-TA)**  **1.3 µM (L-TA)** | **This work** |

**References**

1 Mirceski, V., Komorsky-Lovric, S. & Lovric, M. *Square-wave voltammetry: theory and application*. (Springer Science & Business Media, 2007).

2 Smith, D. E. The enhancement of electroanalytical data by on-line fast Fourier transform data processing in electrochemistry. *Analytical Chemistry* **48**, 517A-526a (1976).

3 Pei, L. *et al.* Formation of Ba bismuthate nanobelts and sensitive electrochemical determination of tartaric acid. *Materials Research Express* **4**, 075047 (2017).

4 Pei, L., Wei, T., Lin, N., Fan, C. & Yang, Z. Aluminium bismuthate nanorods and the electrochemical performance for detection of tartaric acid. *Journal of Alloys and Compounds* **679**, 39-46 (2016).

5 Chen, H. *et al.* Polyaniline/Ba bismuthate nanobelts for sensitive electrochemical detection of tartaric acid. *International Journal of Electrochemical Science* **15**, 1742-1756 (2020).

6 Motia, S., Bouchikhi, B., Llobet, E. & El Bari, N. Synthesis and characterization of a highly sensitive and selective electrochemical sensor based on molecularly imprinted polymer with gold nanoparticles modified screen-printed electrode for glycerol determination in wastewater. *Talanta* **216**, 120953 (2020).

7 Cai, Z. *et al.* CuGeO3/polyaniline nanowires and their electrochemical responses for tartaric acid. *Measurement Science and Technology* **23**, 115701 (2012).

8 Lourenço, A. S. *et al.* Voltammetric determination of tartaric acid in wines by electrocatalytic oxidation on a cobalt (II)-phthalocyanine-modified electrode associated with multiway calibration. *Analytica Chimica Acta* **1008**, 29-37 (2018).

9 Pei, L., Wei, T., Lin, N., Liu, H. & Cai, Z. CuS modified glassy carbon electrode for the electrochemical determination of tartaric acid. *Journal of Bionanoscience* **9**, 239-244 (2015).
